# Development and Validation of the Amphetamine-Type Stimulants Motive Questionnaire in a Clinical Population

**DOI:** 10.3389/fpsyt.2017.00183

**Published:** 2017-09-25

**Authors:** Daniela Thurn, Emmanuel Kuntsche, Jennifer Anna Weber, Jörg Wolstein

**Affiliations:** ^1^Clinical Addiction Medicine, Bezirkskrankenhaus Bayreuth, Bayreuth, Germany; ^2^Department of Psychology, Otto-Friedrich-University Bamberg, Bamberg, Germany; ^3^Centre for Alcohol Policy Research, La Trobe University, Melbourne, VIC, Australia; ^4^Behavioural Science Institute, Radboud University, Nijmegen, Netherlands; ^5^Institute of Psychology, Eötvös Loránd University, Budapest, Hungary

**Keywords:** amphetamine-type stimulants, confirmatory factor analysis, motives for ATS use, scale development, clinical population

## Abstract

Approximately 35.7 million people world-wide use amphetamine-type stimulants (ATS) leading to a high demand for effective treatment. Understanding the motives behind ATS use is a necessary basis for preventive and therapeutic treatment. The objective of this study is to develop the Amphetamine-Type stimulants Motive Questionnaire (AMQ) and to confirm its construct and concurrent validity in respect to the first and the latest month of ATS use based on answers of 233 patients with ATS disorders (74.2% male; mean age: 31.1 years). Confirmatory factor analyses were employed to test for the construct validity of the AMQ. Nested models of confirmatory factor analyses with increasing constraints for gender and age were estimated to test the equivalence of the factor structure in different subgroups. Independent sample *t*-tests were conducted to test for mean differences in the motive dimensions. A structural equation model was estimated to confirm the concurrent validity using the latent four motive factors (i.e., enhancement, coping, social, and conformity motives) as independent variables and frequency of ATS use in the first and the latest month of use as a dependent variable. The results confirmed the AMQ’s four-dimensional factor structure in general, and across gender and age groups for both periods of time. Men (first month: *M* = 4.21, SD = 0.75; latest month: *M* = 3.86, SD = 0.93) use ATS more frequently due to enhancement motives than women (first month: *M* = 3.85, SD = 1.12; latest month: *M* = 3.46, SD = 1.29) at both periods of time [first month: *t*(77) = −2.33, *p* = 0.022; latest month: *t*(80) = −2.19, *p* = 0.031]. Structural equation modeling confirmed an association between coping motives and use frequency, for both periods of time (first and latest month: β = 0.32, *p* < 0.001), as well as between social motives and frequency of use for the latest month of use (β = 0.30, *p* < 0.01). To conclude, the AMQ is a valid and reliable instrument for assessing motives of ATS use in a clinical population. It can provide important insights into the motivational structure of the first and latest months of ATS use which are useful for preventive and therapeutic treatments as well as the development of abstinence skills.

## Introduction

According to the United Nations Office on Drugs and Crime (1), more than 29 million people suffer from drug use disorders. Amphetamine-type stimulants (ATS) comprising substances such as Speed and Crystal Meth have come into focus due to the high potential for drug dependence, cardiovascular and cerebrovascular diseases, infectious diseases or deteriorating dental health, as well as cognitive impairments, depression or psychosis (2–6). ATS are used by approximately 35.7 million people—second only to cannabis with 183 million estimated users, and global seizures reached 173 tons of ATS in 2014 (1). ATS use is particularly common between the ages of 20 and 40 (7, 8) with men being more frequently ATS dependent than women (7). Recent research has investigated a number of ATS-related issues including the illicit use of prescription stimulants (9–11) and the influence of expectancies of stimulant use on cognitive enhancement and the initiation and maintenance of substance use (12–14). Treatment demand has been persistently high since 2003 (1) illustrating the need for effective prevention strategies and treatment measures. To this end, a profound understanding of the motives for ATS use is required. Exploratory studies have made important first steps in this direction but have been restricted by small samples or qualitative data, making a generalization of the results difficult.

Milin et al. (15) investigated different motives of ATS use in outpatient and inpatient settings, albeit without a theoretical basis. They found the motives “I like the effect,” “craving,” “enjoying leisure time,” “mood improvement,” and “going out despite exhaustion” were the most highly rated. Due to the lack of a theoretical model, it however remains difficult to deduce clear recommendations for prevention or treatment strategies. Further studies dealing with ATS use have provided, amongst others, motives such as enhancing sexual performance (15–17), improving (cognitive) performance, performing better or enhancing the efficiency of an action (10, 17–21) and losing weight (15, 17). For persons with attention deficit hyperactivity disorder (ADHD), stimulants cause in addition often paradoxical effects such as calming down and an improved ability to focus on activities (22) and can to some extent be regarded as negatively reinforcing by reducing stressful effects of their activities, tasks and requirements. Other theory-based studies investigated use motives based on the Motivational Model of Alcohol Use (23, 24) for MDMA use (25) and for substance use of psychotic patients (24). To the best of our knowledge, to date there are no instruments that assess the motives for ATS use in a valid and reliable way which are based on a theoretical model. Such an instrument, however, is essential in generating targeted preventive and therapeutic treatments (26–28).

The theoretical framework developed in the Motivational Model of Alcohol Use (23, 29) is a promising approach for classifying the motives for ATS use from a theory-guided emotional-change perspective and laying a foundation for preventative and therapeutic treatments. According to this two-dimensional model, people display certain behaviors in order to achieve a desired affective change (30). This represents the first dimension, valence, with positive reinforcement (i.e., to increase positive feelings) or negative reinforcement (i.e., to decrease negative feelings). The second dimension consists in the source of the affective change which can be internal or external. The crossing of the two dimensions results in the four broad motive categories enhancement, coping, social, and conformity shown in Table 1.

**Table 1:** Classification of (drug) consume motives.

		Valence	
		Positive reinforcement	Negative reinforcement
Source	Internal	Enhancement motives	Coping motives
	External	Social motives	Conformity motives

While originally developed for alcohol use, adaptions of the Motivational Model were successfully transferred to other domains of human functioning such as gambling [GMQ (31)], sexual risk-taking behavior (32), Internet use [IMQ-A (33)], and listening to music [MLMQ (34)]. In the same vein, this article aims to develop a questionnaire to assess motives for using illicit ATS—the Amphetamine-type stimulants Motive Questionnaire (AMQ).

Based on Cox and Klinger’s Motivational Model (23, 30, 35), the Drinking Motive Questionnaire Revised (DMQ-R) was developed which was later published in a short version [DMQ-R SF (36)]. We decided to use the short form of this questionnaire as the basis for the development of the AMQ as it performs very similarly to the longer version DMQ-R (36, 37) but is more viable in a clinical setting. To this end, we adapted the phrasing of the 12 items of the DMQ-R SF to ATS use and converted them into the first person, e.g., “…because you like the feeling” was modified to “…because I like the feeling.” Recent research (15, 38) and clinical observations have provided indications for differences between motives at the beginning of ATS use and after the development of ATS dependence. Therefore, we studied the motives in the first and latest month of ATS use unlike other studies which usually investigate the motives for other behaviors such as alcohol use (32, 36) during the last twelve months. However, in both cases the formulation of all items remained the same as in the DMQ-R SF.

The following hypotheses were tested: (a) For both periods of time, we predicted a good fit of the AMQ four-factor model and high factor loadings of the corresponding items, and at least satisfactory internal consistencies, which provide evidence for construct validity (39, 40). (b) We also predicted, for both periods of time, equivalence of the factor structure among subgroups (gender and age) in respect to the four-dimensional factor structure of the AMQ which is a pre-requirement for group comparisons. (c) Furthermore, we tested mean differences in the subgroups gender and age in the four factors for both periods of time. Based on previous research (38), we expected a higher level of coping motives for women than men. Moreover, we also explored age differences in ATS use motives (due to non-existing evidence in previous studies). (d) Finally, we estimated a multivariate structural equation model to confirm the concurrent validity. Clinical observations and results in the field of alcohol and cannabis use motives (24) have suggested that the use frequency of ATS use (for the first and the latest month of use) is associated with coping and enhancement motives.

## Materials and Methods

### Study Design

We collected data from individuals with ATS disorders who presented as outpatients at drug counseling centers or as inpatients in hospital based drug treatment programs. Of the 133, 11 contacted drug-counseling centers (participation rate: 8.3%), 1 of the 13 contacted cessation therapy clinics specialized in crystal meth addiction (participation rate: 7.7%), 2 contacted addiction departments, and 1 forensic department of Regional Psychiatric Hospitals took part in the study. The Ethics Council of the University of Bamberg granted permission to conduct the study (July 28, 2014/June 06, 2016).

Only patients who were primarily addicted to illicit ATS were asked whether they were interested in participating in the study. The patients were briefed on the goals and contents and were informed about their right to withdraw from the study at any time. Participation was voluntary for all patients and anonymity was guaranteed. The participants were asked to complete questionnaires on their ATS use motives (AMQ), patterns of ATS use and demographic data. All outpatients received a 5€ remuneration for taking part in the study. The data were collected between January 2015 and July 2016.

### Sample and Missing Values

The original sample consisted of 248 individuals with ATS disorders. Participants who did not use ATS as their primary drug of choice (*N* = 8, 3.2%) were excluded as were those who failed to answer more than half of the questions on ATS use motives (*N* = 6, 2.4%) and one participant who took part in the study on two occasions (0.4%). The remaining data included 233 participants. There were no missing values on gender or age. The full information maximum likelihood (FIML) option of the statistical software AMOS (41) was used to account for the remaining missing values (up to 2.1% per item). The analyzed data consisted of 173 men (74.2%) and 60 women (25.8%). The mean age was 31.1 years (SD = 7.9; age range 18–54). Drug counseling centers provided 113 data sets (48.5%), 49 data sets (21.0%) were provided by addiction departments of Regional Psychiatric Hospitals, 43 (18.5%) by a cessation therapy clinic, and 28 (12.0%) by a forensic department of a Regional Psychiatric Hospital.

### Measures

The 12 motive items in the AMQ were adapted from the DMQ-R SF (36) with 3 items per dimension (enhancement, social, coping, and conformity). As the use motives seem to differ between the beginning of ATS use and, e.g., after the development of an ATS addiction (15, 38) the participants were asked to specify how often they used ATS for two periods of time: in their first month of use and their latest month of use. Possible answers were “never” (coded as 1), “seldom” (coded as 2), “sometimes” (coded as 3), “most of the time” (coded as 4), and “always” (coded as 5). The phrasing of all items is given in Table 2.

**Table 2:** Item factor loadings, item means, inter-factor correlations and internal consistencies as results of the confirmatory factor analysis to test the four-factor structure (first month of use/latest month of use).

	Enhancement	Coping	Social	Conformity	Means (SD)
“I used my main drug in the first month vs. in the latest month of use …”					

	First month/latest month of use				

Because I like the feeling	0.61/0.83				4.47 (0.90)/3.97 (1.20)
To get high	0.76/0.54				3.71 (1.28)/3.93 (1.29)
Because it’s fun	0.83/0.79				4.20 (1.03)/3.40 (1.41)
Because it helps when I feel depressed or nervous		0.76/0.76			3.15 (1.33)/3.70 (1.30)
To cheer up when I am in a bad mood		0.75/0.84			3.33 (1.27)/3.82 (1.20)
To forget about my problems		0.73/0.79			3.19 (1.45)/3.85 (1.33)
Because it helps me to enjoy a party			0.81/0.87		3.62 (1.30)/2.57 (1.48)
Because it makes social gatherings more fun			0.48/0.63		3.63 (1.28)/2.90 (1.43)
Because it improves parties and celebrations			0.95/0.88		3.50 (1.37)/2.65 (1.43)
To be liked				0.84/0.78	2.02 (1.20)/1.63 (1.00)
To fit in with a group I like				0.71/0.75	1.91 (1.25)/1.42 (0.83)
So I won’t feel left out				0.85/0.63	1.96 (1.20)/1.75 (1.15)

Correlation with the factor “Coping”	0.16/0.36*				
Correlation with the factor “Social”	0.50*/0.57*	0.06/0.19*			
Correlation with the factor “Conformity”	0.11/0.19*	0.17*/0.18*	0.19*/0.29*		

Internal consistencies (Cronbach’s α)	0.72/0.72	0.79/0.79	0.78/0.84	0.85/0.75	

*Standardized factor loadings that are all significant at the 0.001% error level; model fit: (a) first month of use: CFI = 0.945; TLI = 0.907; RMSEA = 0.071; (b) latest month of use: CFI = 0.940; TLI = 0.898; RMSEA = 0.077*.

*p < 0.05.

The patient questionnaire is based on the “Deutscher Kerndatensatz Klienten (KDS-K) zur Dokumentation im Bereich der Suchtkrankenhilfe” (German core data set of clients for documentation in the field of addiction treatment) (42) which has been applied in German drug-counseling centers and in inpatient drug treatment centers since 2007. It provides, amongst others, information about the age, gender (1 = female, 2 = male) and frequency of use in the first and the latest month of use, coded as monthly frequency, i.e., several times a day = 60, once a day = 30, three to six times a week = 19, one to two times a week = 6, two to three times a month = 3, once a month = 1, and less than once a month = 0.

### Statistical Analysis

We used confirmatory factor analysis (CFA) estimated in statistical software SPSS (AMOS Version 23), in order to confirm the assumed four-factor structure of the AMQ (a) for ATS use in the first month and ATS use in the latest month, separately. Since the formulation of two item pairs was more similar than the third item used to constitute a given latent factor (e.g., the two items “to get high” and “because it’s fun” as enhancement motives) the errors of the two item pairs were allowed to correlate as to compensate answer tendencies (43). We used the Comparative Fit Index (CFI), the Trucker Lewis Index (TLI), and the Root Mean Square Error of Approximation (RMSEA) to assess the model fit. The CFI and TLI compare the tested model with a null or independent model; recommended thresholds for a good model fit are a CFI and a TLI of ≥0.95 (44). The RMSEA represents the mean deviation of the data from the model per degree of freedom. A RMSEA of <0.1 (44) is desirable. To evaluate the internal consistencies of the AMQ, we used Cronbach’s Alpha. Values of 0.7 are considered as satisfactory, of 0.8 as good and of 0.9 as excellent (40).

To test the equivalence of the factor structure in different subgroups (b), we estimated nested models of confirmatory factor analyses with increasing constraints for gender and age (median split: younger users up to 30 years vs. older users over 30 years). First, we tested the configural invariance for the grouping variables gender and age which is supported if the unconstrained model has an acceptable fit to the data. Second, we tested the metric invariance for the grouping variables which requires equivalent factor loadings between the groups (λ-constrained model). Furthermore, we analyzed whether the fit indices remained equivalent when we constrained the variances in addition to the factor loadings (third model) and, finally, when we constrained the factor loadings, the variances and the correlations between the groups (fourth model) (43, 45). We compared the CFI, the TLI and the RMSEA of the fixed models with the fit indices of the freely estimated models.

Furthermore, independent sample *t*-tests conducted in SPSS 23 were used to test mean differences in the motive dimensions (c) between the gender and age subgroups. Therefore, we computed mean scores for the four motive dimensions.

To confirm the concurrent validity (d), we estimated a structural equation model with the latent four motive factors as independent variables and the ATS use frequencies of the first and the latest months of use as dependent variables. All analyses were conducted with SPSS AMOS Version 23 (41) by using the FIML option of AMOS.

## Results

### Confirming Construct Validity of the AMQ

For the first month of ATS use, the CFA provided highly significant factor loadings reaching from 0.48 to 0.95 (all *p* < 0.001; Table 2). The social factor had the lowest and the highest item loadings. The conformity factor had consistently high item loadings from λ = 0.71 to λ = 0.85 with an internal consistency of 0.85. All other internal consistencies were 0.72 or higher. Except between enhancement and social, all interfactor correlations were smaller than 0.20. There was a good fit to the data with a CFI of 0.945, a TLI of 0.907, and a RMSEA of 0.071.

The CFA of the latest month of use also provided highly significant factor loadings (all *p* < 0.001; Table 2) reaching from 0.54 to 0.88. The enhancement factor had the lowest item loading (λ = 0.54) with an internal consistency of α = 0.72. The highest item loading was found on the social factor (0.88), its internal consistency was α = 0.84. As in the first month of use, we observed the highest correlation between the enhancement and the social factor; all others were smaller. There was a good fit to the data with a CFI of 0.940, a TLI of 0.898 and a RMSEA of 0.077.

### Testing Construct Validity in Different Subgroups

In order to test the four-factor structure of the four-factor model according to gender and age groups, we performed nested models of confirmatory factor analyses with increasing degrees of freedom for the subgroups. For the first month of ATS use configural invariance was given for all subgroups (unconstrained models) with CFI and TLI values above 0.9 and RMSEA below 0.1 (Table 3). Metric invariance was also given as the model fit only differs slightly between the unconstrained and λ-constrained models. Furthermore, the fit indices in the two subgroups did not change considerably when we constrained the factor loadings and variances (third model), or the factor loadings, variances and correlations (fourth model). All CFI and TLI values were above 0.9 and all RMSEA values below 0.1.

**Table 3:** Model fit according to gender and age (first month of use and latest month of use).

	χ^2^	df	CFI	TLI	RMSEA
	
	First month/latest month of use				
**Gender (men vs. women)**					
Unconstrained model	140.2/150.2	92	0.949/0.944	0.914/0.906	0.048/0.052
λ-Constrained model	154.1/170.6	100	0.943/0.933	0.911/0.895	0.048/0.055
Plus variance constrained	162.9/184.5	104	0.938/0.923	0.907/0.885	0.050/0.058
Plus correlations constrained	168.1/195.9	110	0.939/0.918	0.913/0.884	0.048/0.058

**Age groups (≤30 vs. >30 years)**					
Unconstrained model	140.5/160.5	92	0.951/0.935	0.916/0.890	0.048/0.057
λ-Constrained model	157.1/176.4	100	0.942/0.928	0.909/0.888	0.050/0.058
Plus variance constrained	161.7/179.2	104	0.941/0.929	0.912/0.893	0.049/0.056
Plus correlations constrained	174.0/186.7	110	0.935/0.928	0.908/0.897	0.050/0.055

For the latest month of ATS use, configural invariance was given in the unconstrained model for the subgroup gender with CFI and TLI values above 0.9 and RMSEA below 0.1 and almost given for the subgroup age with a CFI value of 0.935, a TLI value of 0.890, and RMSEA below 0.1. All other tested models (λ-constrained model, plus variance constrained model, and plus correlations constrained model) displayed similar CFI values above 0.9, TLI values very close to 0.9, and RMSEA values below 0.1 in both subgroups gender and age (Table 3).

The results basically remained the same when the sample was split at age 25 and 35, respectively. The resulting model fit indices can be obtained from the authors on request.

### Testing Mean Differences between the Subgroups

In the first month of ATS use, men scored higher on positive reinforcement (enhancement and social motives). However, this difference was only significant for enhancement motives (Table 4). Women scored somewhat higher on negative reinforcement (coping and conformity motives) but the differences were not statistically significant. The subgroup age revealed no significant differences in the four motive dimensions. Younger ATS users scored slightly higher on enhancement, social, and conformity motives and lower in coping motives than older ATS users but none of these differences were significant.

**Table 4:** Means (SDs in brackets) of the four motive dimensions according to gender and age group and independent sample *t*-tests (first month and latest month of use).

	Enhancement	Coping	Social	Conformity
	
	First month/latest month of use			
**Gender**				
Women	3.85 (1.12)/3.46 (1.29)	3.40 (1.20)/3.79 (1.28)	3.40 (1.11)/2.52 (1.19)	1.99 (1.18)/1.50 (0.77)
Men	4.21 (0.75)/3.86 (0.93)	3.17 (1.12)/3.79 (1.04)	3.64 (1.10)/2.77 (1.21)	1.95 (1.02)/1.64 (0.83)
*t* Value	−2.33*/−2.19*	1.32/0.01	−1.44/−1.41	0.29/−1.14

**Age group**				
≤30 years	4.17 (0.91)/3.87 (1.06)	3.21 (1.22)/3.89 (1.10)	3.67 (1.05)/2.79 (1.23)	2.02 (1.10)/1.60 (0.78)
>30 years	4.06 (0.84)/3.64 (1.02)	3.25 (1.05)/3.69 (1.11)	3.48 (1.15)/2.62 (1.19)	1.90 (1.02)/1.60 (0.85)
*t* Value	0.86/1.66	−0.24/1.35	1.33/1.08	0.83/−0.03

Total	4.12 (0.87)/3.76 (1.04)	3.23 (1.14)/3.79 (1.11)	3.58 (1.10)/2.70 (1.21)	1.96 (1.06)/1.60 (0.82)

*p < 0.05.

In the latest month of use, men scored higher on positive reinforcement (enhancement and social motives) and on conformity motives with a significant difference in enhancement motives (Table 4). Women and men scored identically on coping motives. Also for the latest month of ATS use, the subgroup age revealed no significant differences in the four motive dimensions.

### Confirming Concurrent Validity of the AMQ

The estimation of the structural equation model revealed that higher coping motives are connected with higher frequency of ATS use for the first and the latest month of ATS use. A further positive association was found between social motives and frequency of ATS use for the latest month of use (Table 5). The explained variance was 12.6 and 15.6% for the first and the latest month of ATS use, respectively. In a final step, the link between the four motive factors and frequency of ATS use was adjusted for gender and age effects. However, the results (not shown but available from the authors on request) remained basically unchanged due to non-significant gender and age effects.

**Table 5:** ATS use frequency (standardized regression coefficients and explained variance).

	Enhancement	Coping	Social	Conformity	*R*^2^
	
	First month/latest month of use				
Use frequency	0.10/−0.14	0.32***/0.32***	0.03/0.30**	−0.02/−0.02	12.6/15.6%

***p < 0.001; **p < 0.01; model fit for the first month of ATS use: CFI = 0.945; TLI = 0.908; RMSEA = 0.066. Model fit for the latest month of ATS use: CFI = 0.936; TLI = 0.893; RMSEA = 0.074.

## Discussion

The aim of this study was to develop the four-dimensional AMQ and to validate it with regards to the first month and the latest month of ATS use. It was expected that the AMQ and its underlying theoretical considerations are valid for both periods of time which was formally tested in this article.

The first aim (a) was to test construct validity (39) by means of CFA which revealed good model fit according to the CFI and the RMSEA values and acceptable model fit according to the TLI values for the hypothesized four-factor model for motives of ATS use. The values for the recall of the first month of ATS use were consistently slightly higher in terms of factor loadings of the corresponding items which means that the data gathered for the first period of time was slightly better approximated by the four-factor model. The highest interfactor correlation was found between social and enhancement for both periods of time with slightly higher values for the recall of the latest month of use. This indicates that those who used ATS for instance “because it is fun” also tended to use it because it improves parties and celebrations. This relation may become even more important over time. A strong link between enhancement and social motives was also found for alcohol use (32, 36) and listening to music (34). Furthermore, consistent with previous research on other domains of human functioning, high internal consistencies were found for both periods of time (40) which is particularly remarkable considering only three items were used to measure each factor.

Second (b), the CFA in the subgroups demonstrated the equivalence of the four-dimensional factor structure of the AMQ across gender and age. This not only indicates that the theoretical assumptions held true in both subgroups but also that the AMQ can be used to compare both subgroups. Based on the different scores in the motive dimensions, subgroup-specific interventions may be deduced for clinical practice.

The analysis of mean differences across subgroups (c) revealed that men use ATS more frequently due to enhancement motives than women. This was true for both periods of time. A possible explanation is that men are generally more open to new experiences, seek extreme sensations, and are more willing to experience adverse consequences (46, 47). No gender-specific differences were observed at either period of time in any other motive dimension. The expected differences in the coping dimension could not be confirmed, which may be explained by the fact that our study includes a different selection of items than the cited study (38). There were no significant age differences in the four motive dimensions for either period of time. Therefore, motive based therapies may be applied to a wide age range.

The questionnaire was found to have concurrent validity (d) as the structural equation model revealed a significant positive relation between coping motives and frequency of use in the first and latest month of ATS use, i.e., those who used ATS to cope used them more frequently. This is to some extent consistent with previous research (24) in which the frequency of ATS use was associated with coping and enhancement motives and underlines the vicious circle in which patients with ATS disorders often end up: coping with problems caused by using ATS leads to further ATS use. Furthermore, there was a significant positive relation between social motives and frequency of use in the latest month of ATS use, i.e., those who used ATS for social reasons in this time period used them more frequently. This may be related to the circle of acquaintances the users have built and with whom they party regularly.

### Limitations and Recommendations for Future Research

Since the study relies on the retrospective recall of use motives, memory bias constitutes an important limitation (48) which may partly explain the sufficient but not optimal model fit. The sample consisted of a heterogeneous group of patients which differed in the length of their abstinence: some presented themselves at drug counseling centers and were not abstinent at that time, some were in cessation therapy clinics and were abstinent from 0 to 6 months and others again were in the forensic department of a Regional Psychiatric Hospital and were abstinent from a few months to a few years. Depending on the recency of their last ATS use (49–51) and history of treatments (52), patients had different opportunities to deal with their relapses, to develop abstinence skills and to reflect on their motives of ATS use. Particularly the latter aspect may have had an effect on the answers given in the questionnaire. While mean levels of motive endorsement may be subject to recall bias, the four-factor classification of motives according to the valence and the source is not affected as studies in the field of alcohol use have shown (37, 45). We, thus, consider the recall bias as reasonable trade-off between feasibility of data collection and accuracy. In addition, some important aspects in the field of ATS use had not been considered in the 12-item AMQ: individuals with ATS disorders reported using ATS to improve sex (15–17), to enhance their cognitive performance (10, 17–21), to lose weight (17), to be able to stay awake, and as a self-medication (22) for symptoms of ADHD. Although the aforementioned reasons seem to be less common compared to items in the AMQ such as getting high or having fun (17), further studies should examine the importance of other amphetamine-specific motives of ATS use. Considering the intricate circumstances in this clinical setting—e.g., approaching ATS users and convincing therapists of the advantages of participating in the study—the sample size of 233 people can be regarded as sufficient. Larger sizes of clinical samples are difficult to achieve. This becomes apparent in the generally low response rates reported in the study design section which may have had an effect on the results. Moreover, it should be examined whether the AMQ, which was developed and validated in this study using a clinical sample in Southern Germany, can also be applied to non-clinical samples and in other countries. It would also be worthwhile to apply the AMQ to primary substances like illicit prescription stimulants or MDMA and related drugs. Furthermore, longitudinal studies are needed to be able to prospectively evaluate the development of ATS motives over time. Finally, we recommend including additional outcome variables in future research to extend the concurrent validity of the AMQ.

### Conclusion

The application of the Motivational Model of Alcohol Use (23, 29) to ATS use is an important step toward a better understanding of why people use ATS particularly since most use the substance in spite of knowing the high potential for dependence and the multiple somatic and mental consequences (2–6). Moreover, as a theory-based instrument, the AMQ closes a gap in ATS research and may assist in generating targeted preventive and therapeutic treatments (26–28) as it can reveal recalled former and current motives of ATS use. For the assessment of use motives of patients, the AMQ can help understand why people begin and continue to use ATS and in a second step to personalize the treatment which has to manage the resulting changes related to ATS abstinence (17). In this study, the AMQ was shown to be succinct and viable in a clinical setting and has demonstrated its construct validity and reliability. It also constitutes a basis for future research, in particular to study changes in the motive structure between the first and latest month of ATS use.

## Ethics Statement

This study was carried out in accordance with the recommendations of the Ethics Council of the University of Bamberg with written informed consent from all subjects.

## Author Contributions

DT formulated the hypotheses, planned and scheduled the study (establishing cooperation with the participating institutions, raising funding), conducted the study (coordinating the gathering of data, acting as a contact person for the participating institutions), analyzed the results statistically, and wrote the article. EK formulated the hypotheses, planned the study, analyzed the results statistically, and wrote the article. JWe formulated the hypotheses, planned the study (establishing cooperation with the participating institutions), conducted the study (acting as a contact person for the participating institutions), analyzed the results statistically, and wrote the article. JWo formulated the hypotheses, planned the study (establishing cooperation with the participating institutions, raising funding), analyzed the results statistically, and wrote the article.

## Conflict of Interest Statement

The authors declare that the research was conducted in the absence of any commercial or financial relationships that could be construed as a potential conflict of interest.
